# Postpartum breast cancer as a distinct oncologic entity: linking physiological tissue remodelling to tumor biology and clinical translation

**DOI:** 10.1186/s13058-026-02341-5

**Published:** 2026-07-08

**Authors:** Vidya P. Nimbalkar, Snijesh V. P., Akash Dash, Meenakshi Jothikumar, Anjaney J. Pandey, P. S. Hari, Aruna Korlimarla, Sabarinathan Radhakrishanan, Jyothi S. Prabhu

**Affiliations:** 1 Division of Molecular Medicine, St. John’s Research Institute, St. John’s Medical College, Bangalore, Karnataka India; 2 National Centre for Biological Sciences, Tata Institute of Fundamental Research, Bangalore, Karnataka India; 3 Department of Molecular Oncology, Sri Shankara National Center for Cancer Prevention and Research, Sri Shankara Cancer Foundation, Bangalore, India

**Keywords:** Postpartum breast cancer, Mammary gland involution, Extracellular matrix remodelling, Immune infiltration, Estrogen signaling, Therapeutics

## Abstract

Postpartum breast cancer (PPBC), defined as breast cancer diagnosed within 10 years after childbirth, is increasingly recognized as a biologically distinct and clinically aggressive subtype affecting young women worldwide. The postpartum mammary gland undergoes extensive remodelling during involution, characterized by inflammation, extracellular matrix reorganization, and immune suppression, collectively creating a tumor-promoting microenvironment. Recent studies have revealed unique molecular features of PPBC, including alterations in immune composition, stromal activation, and selective pathway enrichment that distinguish it from breast cancers in nulliparous or age-matched parous women. While estrogen signalling remains an important regulatory axis during the postpartum period, its interplay with involution-associated processes and tumor behaviour requires further elucidation. In this review, we summarize current advances in understanding PPBC biology, highlight emerging biomarkers, and discuss evolving therapeutic considerations relevant to this high-risk population. We also provide an overview of ongoing clinical investigations, such as aspirin-based anti-inflammatory trials aimed at targeting postpartum-specific mechanisms that may contribute to tumor initiation or progression. Together, these insights emphasize the need for dedicated translational research and tailored clinical strategies to improve early detection, risk stratification, and outcomes for women diagnosed with PPBC.

## Introduction

Breast cancer (BC) is the most commonly diagnosed cancer in women worldwide, and ~ 2.3 million new cases and ~ 670,000 deaths reported in 2022 [1], with an increasing number of cases diagnosed each year. Though BC is considered an elderly women disease, incidence of BC in younger women (< 45 years) is increasing [2]. BC in young women is more aggressive and associated with poor prognosis [3–5]. Studies report that this might be due to the fact that young women do not undergo routine screening and present the disease at later stages. Young women BC accounts for nearly 13% of all BC in developing countries [6] and the percentage is higher in lower economic countries [7–9]. Around 50% of the BC in young women are developed within 5–10 years of recent childbirth highlighting the postpartum period as a crucial window of susceptibility to develop BC [6, 10–12]. Postpartum breast cancer (PPBC) is defined as BC diagnosed within 5–10 years after childbirth [10, 13]. There are inconsistencies across the studies in defining the risk period for developing PPBC, ranging from 5 years to 15 years after delivery [13–15]. However, all the studies are in concordance that risk is higher during first 5 years. Earlier studies have considered PPBC as pregnancy associated breast cancer (PABC) [16, 17]. The definition of PABC varies, being described either as BC diagnosed only during pregnancy or as including cases identified within 6–12 months after childbirth and the incidence of PABC is much lower than PPBC [18–20]. A study by Frédéric Amant et al. reported that, although pregnancy and the postpartum period are closely linked, BC during pregnancy (PrBC) and PPBC are distinct entities, as they differ in biology and prognosis. Notably, PPBC may extend up to 5–10 years after childbirth and is more aggressive, with a poorer prognosis than PrBC [21]. PPBC patients show significantly poorer prognosis compared to age, stage, and biologic subtype matched nulliparous BC patients or parous BC patients who are more than 10 years postpartum [10, 15]. Studies have reported that in PPBC patients, five years distant metastasis-free survival (DMFS) rates can be as low as 70%, and ten years DMFS falls to 50% [10, 15]. Notably, this poor prognosis of PPBC is consistently observed across multiple geographic regions, including North America, Europe, Asia, and Africa, highlighting its global clinical relevance and reinforcing the need for improved risk stratification and management [17, 22–28]. Despite being more aggressive and a distinct disease, there is currently no treatment design to target this aggressive subtype of BC. Currently, these patients receive the same subtype-specific treatments as those used for other BC patients. PPBC affects young women who are in the phase of establishing their careers and families and this disease impacts their lives both physically and psychologically. This creates a huge emotional and financial burden on society. Thus, there is an urgent need to understand the biology of PPBC for better management of the disease and developing better therapeutic strategies. This review explores the unique pathobiology of PPBC and possible therapeutic options that could be developed.

### Epidemiology

Worldwide incidence of PPBC is approximately 130,000–350,000 cases each year [6]. Epidemiological studies further show that the peak incidence of BC occurs between 40 and 50 years in Asian countries, whereas in Western populations the peak is observed later, between 60 and 70 years [29, 30]. BC strikes Asian women earlier than it does Western women [30, 31]. A similar trend also observed in several African countries showing higher burden of young onset breast cancer (YOBC) and consequently lower burden of BC in postmenopausal women [32]. Though these geographic differences may be influenced by younger population structures, it also shows the BC biology across diverse populations. Recent studies evaluating the clinicopathological features of PPBC tumors have demonstrated that the metastatic nature of PPBC is independent of molecular subtype, tumor size, grade, or even Ki-67 expression levels [15]. PPBC tumors within  5 years of childbirth are known to have higher rates of lymphovascular invasion and increased lymph node involvement compared to age-matched nulliparous or parous BC patients more than 5 years of childbirth [33].

Parous women have a 10–30% higher risk of developing BC within a decade after childbirth in comparison with nulliparous young women [6, 34]. Age at diagnosis, parity status, and breastfeeding history are primary risk factors associated with PPBC [15, 35]. One of the most consistently reported risk factors for PPBC is older maternal age at first delivery. Women who give birth to their first child at or after age 30 have a higher likelihood of developing PPBC compared to those who deliver in their twenties, suggesting that parity-related protection diminishes with delayed first childbirth [34]. Similarly, age at last delivery also significantly influences the risk of PPBC [35]. This trend may explain rising BC incidence rates globally due to delayed childbearing in developed countries [15, 35, 36]. The relationship between parity and PPBC is complex and non-linear. Although higher parity generally correlates with reduced long-term BC risk, this protective effect is modulated by maternal age at the time of delivery [37]. Women who have one or two children in their 30s have a greater risk of developing BC than those who have children in their 20s [38]. The inter-pregnancy interval further modifies risk. A delivery gap under five years has been associated with higher BC risk than a gap longer than five years [39].

Beyond reproductive history, breast tissue characteristics also contribute to PPBC development. Women with higher breast density have approximately 2.4 times greater BC risk than those with lower density [40, 41]. High breast density reflects increased fibroglandular tissue, which includes both stromal and epithelial components, and has been associated with an environment that may influence BC risk [42]. Genetic predisposition also modifies risk. Among BRCA1 pathogenic variant carriers, those diagnosed with PPBC within 5–10 years postpartum exhibit a two-fold increase in mortality, whereas no such correlation is observed for BRCA2 carriers [43].

### Pathophysiology of PPBC

#### Postpartum mammary gland involution

During weaning, the mammary gland undergoes remodelling to regain its pre-pregnant form; this remodelling process is called involution [44]. Involution occurs in two major phases [45]. The first phase is acute and reversible, while the second phase is irreversible and characterized by epithelial cell death, immune infiltration, and tissue remodelling, along with the differentiation and repopulation of stromal cells by adipocytes [46]. Cell death in the first phase of involution is proteinase-independent, whereas the irreversible second phase uses matrix metalloproteinases (MMPs) and serine proteases, and is therefore referred to as the proteinase-dependent phase [46]. Short duration of breastfeeding leading to abrupt involution has been associated with changes in the mammary microenvironment that may be consistent with an increased risk of BC [47]. Significant pro-tumorigenic changes observed in mouse mammary glands following abrupt, but not gradual, involution demonstrate the loss of the potential protective benefits of prolonged breastfeeding in parous women [47]. Abrupt involution causes a chronic inflammatory microenvironment by accelerating cell death, DNA repair response, and immunosuppressive myeloid cell infiltration, while gradual involution shows a more controlled immune response [48].

#### First phase of involution

**Fig. 1:**
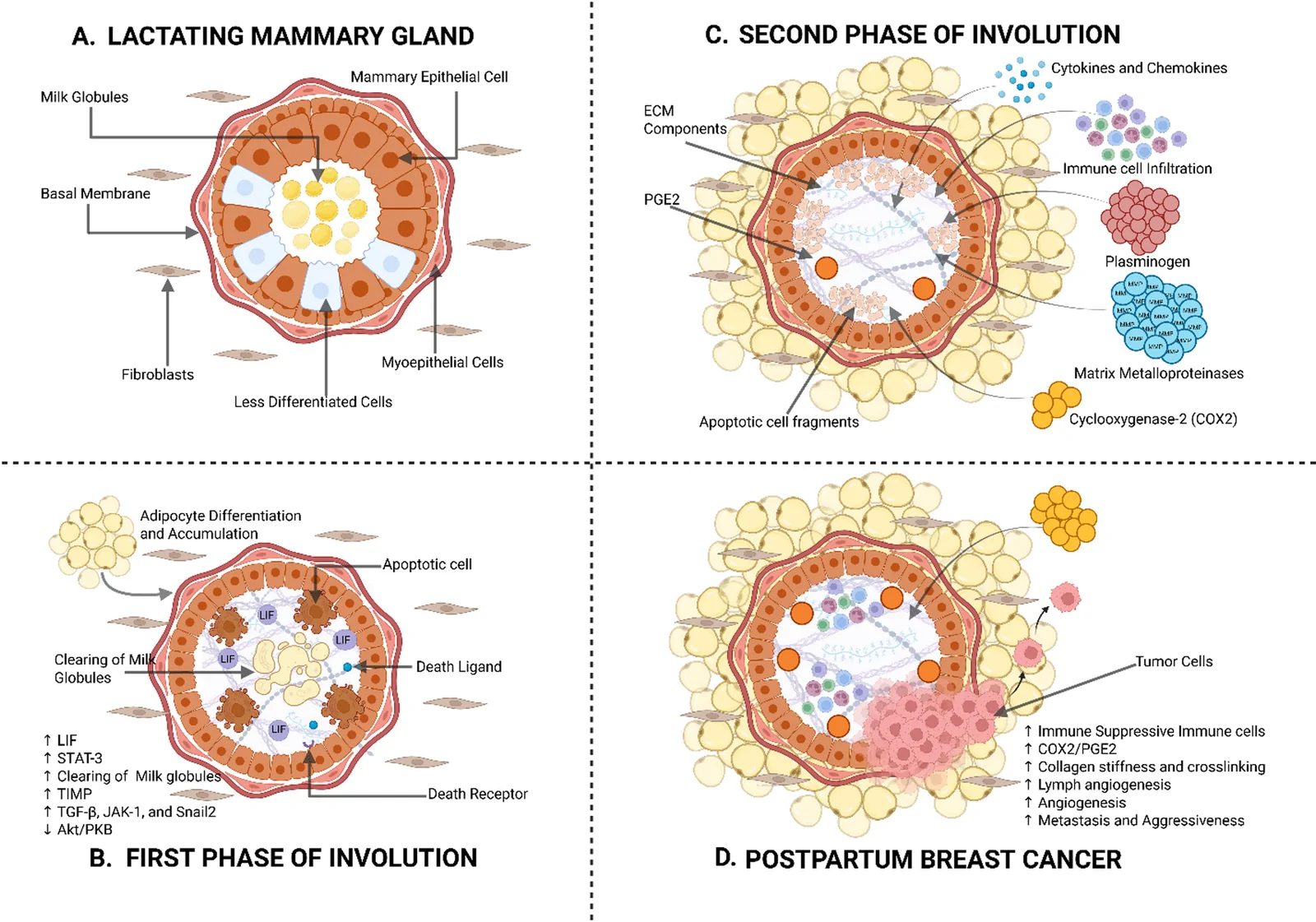
Phases of Involution and PPBC **A** lactating mammary gland; once the mother starts weaning the mammary gland undergoes changes to revert to the pre-pregnancy state in a process called involution. Involution is a two-step process, **B** First phase of involution starts off with clearing of milk globules and LIF mediated STAT3 regulated apoptosis of the mammary epithelial cells, STAT3 is kept active by the activation of LIF and TGF-β. **C** Second phase of involution is an irreversible process due to the influx of MMPs, this phase undergoes ECM remodelling as well as immune cell infiltration. Amongst all this, MMPs and plasminogen help cleave and reshape the ECM, while cytokines, chemokines along with COX2 mediate inflammation. **D** PPBC occurs through dysregulation in the process of involution causing an imbalance that cascades into PPBC. During lactation, epithelial cells produce milk and adipocytes shrink, in the process of involution epithelial cells die due to apoptosis and adipocytes expand to fill the gland. During involution, stromal cells (fibroblasts and immune cells) are involved in remodelling the tissue, that creates a wound healing state. Dysregulated involution leads to increase in the tumor associated macrophages (TAMs), cancer associated fibroblast and ECM stiffening. (Created using BioRender.com)

The initial phase of involution occurs within the first 48 h after weaning. It is characterized by milk stasis, where milk accumulates in the ducts due to the cessation of sucking. During this period, prolactin and glucocorticoid signaling are downregulated and STAT3-driven apoptosis plays a central role [49]. The cytokine leukemia inhibitory factor (LIF) is a key upstream regulator of STAT3 with its expression increasing nearly 30-fold within the first 12 h. Without STAT3 activation, epithelial cell death is suppressed and involution halts [50, 51]. Li et al. demonstrated that milk stasis leads to STAT3 phosphorylation, triggering mammary-gland involution via lysosomal-mediated cell death rather than classical apoptosis [52]. TGF-β is also known to regulate STAT3 activation at the onset of involution [53]. Mechanistically, STAT3 shifts the balance toward cell death by modulating BCL2-family members and inhibiting PI3K signaling [54, 55]. STAT3 drives an initial, caspase-independent lysosomal cell death pathway through upregulation of cathepsins and downregulation of the endogenous cathepsin inhibitors [56]. During normal involution, following the initial cell death and clearance, involution transitions to the second phase. However, dysregulation of these pathways may result in chronic or dysregulated STAT3 signaling, allowing some cells to escape death and potentially initiate or promote tumorigenesis [57]. Key molecular and cellular changes that happen in the lactating mammary gland and during first phase of the involution are represented in Fig. 1A and B respectively.

#### Second phase of involution

The second-phase transition happens approximately after 48 h. During this stage tissue architecture of the gland undergoes irreversible tissue remodelling. The architecture of gland undergoes significant reorganization with adipocytes repopulating the alveolar spaces leading to alveolar collapse. These processes are largely driven by MMPs and serine proteases which are considered primary regulators of extracellular matrix (ECM) degradation, cell death, and stromal remodelling [58]. Stromal cells express MMPs, notably MMP-2, MMP-3, and MMP-9, which are upregulated during involution [59]. Cells that survived the first phase might undergo detachment induced apoptosis. MMP-3 does not regulate mammary epithelial cell death but regulates adipogenesis during mammary gland involution [60]. Expression of MMP-2 is tightly regulated by tissue inhibitors of metalloproteinases (TIMPs). MMP-2 primarily degrades ECM components such as type IV and V collagen, fibronectin, laminin, and elastin. Dysregulation of MMP-2 is frequently associated with tumor progression [61]. MMP-9, a gelatinase in the same subfamily as MMP-2, contributes to detachment induced apoptosis by cleaving ECM and adhesion molecules [62]. MMP-2 and MMP-9 are upregulated, while TIMP-1 is downregulated during adipocyte differentiation. The plasminogen cascade of serine proteases plays an important role in mammary gland development [63]. Plasminogen is activated to plasmin by urokinase-type plasminogen activator (uPA), tissue-type plasminogen activator (tPA), and plasma kallikrein (PKal), with plasma kallikrein acting as the dominant activator in this context. MMP-2 and MMP-3 are low during lactation and in the initial days of involution, but by day 4 they are strongly upregulated alongside the serine proteinase uPA. Absence of plasminogen delays involution and leads to abnormal adipocyte differentiation [59]. A study has demonstrated that mice lacking plasminogen exhibit incomplete mammary gland involution likely to compromise its ability to support subsequent lactation [59]. Beyond proteases, mammary-epithelial-cell (MEC) like macrophages play an important role in engulfing apoptotic cells, leading to the release of anti-inflammatory mediators that suppress pro-inflammatory signals. Clearance of apoptotic cells by MEC like macrophages is therefore critical for tissue homeostasis and the resolution of inflammatory lesions [64]. While all this happens, MMPs and serine proteases participate in shaping the stroma by depositing stromal proteins such as collagen and many glycoproteins to revert the mammary gland architecture to that of its pre-pregnancy state. Key molecular and cellular changes are represented in Fig. 1C. The changes that happen in the ECM during this irreversible phase are discussed below.

### Extracellular matrix remodelling from normal involution to postpartum breast cancer and its progression

Postpartum mammary gland involution is a highly dynamic and tightly regulated process characterized by extensive ECM remodelling that transiently creates a wound healing like microenvironment. Many of the ECM changes observed during involution are similar to those seen in BC, and this may create a unique window of vulnerability during which physiological tissue remodelling could potentially support tumor initiation. During normal involution, the mammary epithelium undergoes programmed cell death accompanied by immune infiltration and substantial ECM remodelling. Proteolytic enzymes, including MMP-2, MMP-3, MMP-9, and the serine proteases uPA, tPA, and plasma kallikrein, degrade key structural components such as laminin, fibronectin, and type IV collagen [65, 66]. These events may facilitate epithelial cell detachment and anoikis, enabling the tissue to regress to its pre-pregnancy state. Importantly, this process also generates ECM fragments that have been shown to support tumor cell growth in vitro, highlighting the potential intrinsic tumor-promoting properties of the involuting microenvironment [67, 68]. Under normal conditions, this wound-like phase resolves, restoring homeostasis; however, when involution is prolonged or dysregulated, the ECM may acquire features similar to those observed in tumor-supportive microenvironments.

Aberrant or delayed involution results in sustained ECM remodelling that promotes fibrosis, increased collagen deposition, and excessive LOX-mediated crosslinking, all of which might contribute to matrix stiffening. In parallel, persistent MMPs activity generates additional ECM fragments [47]. This might lead to elevated levels of pro-inflammatory cytokines such as IL-6, TNFα, and TGF-β that maintain a chronic inflammatory environment. These inflammatory and mechanical alterations, together with enhanced angiogenesis and lymphangiogenesis, create a highly conducive environment for tumor initiation. The ECM in the postpartum breast comprises of the basement membrane that is rich in laminin, collagen, fibronectin, entactin, glycoproteins proteoglycans, and hyaluronic acid [69, 70]. Many of these components may actively promote tumor progression and metastasis [71]. Hyaluronic acid further contributes to ECM remodelling and may enhances tumor aggressiveness, particularly in more invasive BC subtypes [72].

As involution proceeds, collagen accumulation and crosslinking may make the ECM denser and mechanically stiffer [72, 73]. Tumor or residual epithelial cells can sense this stiffness primarily through integrins, which transduce extracellular mechanical forces into intracellular signals via focal adhesion complexes involving FAK, Src, and associated cytoskeletal regulators [72, 74]. Activation of these pathways has been associated with cytoskeletal reorganization, motility, and survival signalling, and may promotes epithelial-to-mesenchymal transition (EMT) through TGF-β and SNAIL dependent mechanisms [71, 75, 76]. Consequently, epithelial cells may lose polarity and adhesion and acquire more motile and invasive phenotypes. Collagen fibers within the remodeled ECM have been reported to align into linear and radially oriented structures. However, long-term activation of TGF-β and PGE2 polarizes macrophages toward an M2 like phenotype, that secretes IL-10, ARG1, and MMP-9, suppressing cytotoxic immunity and promoting collagen deposition and ECM stiffening (TACS-3) structures that have been associated with directional tumor cell invasion [37, 77, 78]. Persistent inflammation, proteolysis, fibroblast activation, and matrix reorganization may collectively support directional migration pathways and facilitate early tumor spread. In the postpartum context, studies suggest that involution may be accompanied by increased lymphangiogenic signaling including elevated VEGF-C and VEGF-D levels. These cues may promote expansion and remodelling of periductal lymphatic vessels, which can be associated with reorganized collagen channels that may increase lymphatic accessibility. Together, these changes may create structural pathways that could facilitate tumor cell dissemination during the postpartum period.

The stiffened and reorganized matrix may also act as an active signaling platform. Collagen-sensing receptors such as DDR1 and DDR2 can detect structural changes in collagen fibrils and may cooperate with hypoxia-induced factors like HIF-1 to promote invasive and pro-metastatic signalling [70]. Pro-inflammatory cytokines such as IL-6, TNFα, and TGF-β can induce MMPs and LOX, potentially contributing to cycle of ECM degradation, crosslinking, and reorganization [79, 80]. These mechanical and biochemical feedback loops are associated with tumor cell proliferation, invasion, angiogenesis, lymphatic remodeling, immune evasion, and therapeutic resistance, and may contribute to microenvironmental changes in the postpartum gland that support tumor progression [76].

Fibroblasts may play a central role in shaping this tumor-permissive ECM. During postpartum involution, mammary fibroblasts can adopt a contractile, myofibroblast-like phenotype characteristic of wound healing [81]. In the context of PPBC, persistent TGF-β signalling has been suggested to promote the transition of these fibroblasts into cancer-associated fibroblasts (CAFs), which can deposit collagen, fibronectin, LOX, and MMPs, potentially contributing to increasing ECM stiffness and architectural complexity [81, 82]. Mechanically stiff ECM has been associated with activation of YAP/TAZ transcriptional regulators, promoting gene programs associated with stemness, proliferation, EMT, and therapy resistance [83, 84]. CAFs near tumor cells may produce dense collagen networks that can facilitate invasion, whereas those more distant may contribute to a broader pro-inflammatory niche through secretion of IL-6 and production of hyaluronic acid [81]. Importantly, ECM-immune crosstalk may also influence PPBC progression. COX2/PGE2 signalling has been reported to program macrophages toward a pro-tumorigenic phenotype that may enhance ECM remodelling and facilitates immune evasion [68]. Semaphorin-7 A (SEMA7A) is a GPI-anchored glycoprotein, that is highly expressed during involution and has been reported to be elevated in PPBC tissues. It has been implicated in activating multiple pathways in PPBC progression, including increasing COX2 expression in tumor cells and promoting collagen deposition by fibroblasts in the surrounding microenvironment [33]. These cellular and structural interactions may contribute to self-reinforcing cycles of inflammation, mechanical stress, and ECM remodelling that could support tumor growth and aggressiveness.

Collectively, the postpartum ECM may contribute to local tumor initiation and could also influence metastatic dissemination. Basement membrane degradation by MMPs may enable tumor cells to rupture tissue boundaries, while aligned collagen fibres may facilitate collective migration. LOX-mediated collagen crosslinking has been associated with the establishment of pre-metastatic niches in distant organs. Lymphangiogenic remodelling may enhance tumor access to lymphatic vessels, and the cytokine-rich microenvironment may recruit immune-suppressive cells such as M2 macrophages. Circulating ECM fragments have also been suggested to influence distant sites for metastatic colonization. These processes suggest that the postpartum breast may become an active participant in shaping the aggressive clinical behavior of PPBC. Dysregulated involution leads to alteration in ECM remodelling leading to development of PPBC. This is depicted in Fig. 1D. Selected studies investigating ECM remodelling, inflammation, and tumor promotion during postpartum mammary gland involution are mentioned in Table 1.

**Table 1:** Selected studies investigating extracellular matrix remodeling, inflammation, and tumor promotion during postpartum mammary gland involution.

Study focus	Experimental model	Mechanistic findings
Collagen and COX2 in tumor progression during postpartum involution [85]	In vivo xenograft: human breast cancer cells injected into involuting vs. nulliparous mouse mammary gland	Involuting mammary glands exhibit elevated fibrillar collagen and COX2 levels, which augment cancer invasiveness Conversely, COX2 inhibition diminishes collagen deposition, tumor proliferation, and metastasis.
Postpartum activated fibroblasts; COX2/PGE2 [73]	Murine model: PDGFRα⁺ fibroblasts isolated from nulliparous or involuting (postweaning) glands; co-injection with syngeneic tumor cells in orthotopic transplants	Involution fibroblasts promote tumor growth through COX2/PGE2 (increasing collagen, LOX, TGFβ1, CXCL12) and establish an immunosuppressive environment NSAIDs restore fibroblast function and diminish tumor promotion.
SEMA7A– mediated invasion and survival in PPBC [33]	Mouse model: syngeneic tumor cells with SEMA7A knockdown or overexpression in postpartum vs. nulliparous hosts	SEMA7A increases tumor growth, invasion, COX2, and collagen deposition in PPBC Silencing decreases tumor burden. High SEMA7A/COX2/fibronectin predicts poor prognosis.
SEMA7A promotes lymphatic remodelling via macrophages during involution [86]	Mouse model: wildtype vs. SEMA7A⁻/⁻ mice; tumor xenografts and macrophage analyses	Involutional SEMA7A promotes lymphangiogenesis and metastasis by inducing PDPN⁺ macrophages, Deletion of SEMA7A lowers PDPN⁺ macrophages and lymphatic remodelling.
LOXL2 in metastasis and involution matrix remodelling [87]	In vitro and mouse models: LOXL2 knockdown in 4T1/ MDA-MB-231 cells (orthotopic xenografts, MMTVPyMT transgenics)	The co-expression of LOXL2, TIMP1, and MMP-9 during involution promotes ECM remodelling and tumor invasion. LOXL2 inhibition diminishes metastasis and collagen crosslinking.
MMP-3 (Stromelysin-1) and TIMP-1 in involution remodelling [60]	Transgenic mouse models: TIMP-1 overexpressor and MMP-3 (Str1) knockout strains	Involution increases MMP-3, which regulates stromal remodelling and adipogenesis MMPs inhibition increases adipocyte lipid accumulation and differentiation.
COX2, MMPs, and ECM remodelling as tumor-promoting characteristics in PPBC [88]	Human breast tissue samples post-weaning analyzed by histology, IHC, and RNA-seq	Breast involution during weaning leads to COX2 and MMP-mediated ECM remodelling, immunological infiltration (CD45⁺, CD68⁺), and TGFB1-driven wound-healing pathways, creating a pro-tumorigenic environment linked to PPBC.
COX2–mediated lymphangiogenesis in facilitating lymph node metastases during postpartum mammary gland involution [68]	Murine postpartum involution model and COX2 knockout mice	COX2 overexpression during involution increases PGE2-mediated VEGF-C production, promoting lymphangiogenesis and nodal metastasis. COX2 inhibition (Celecoxib) inhibits both processes, linking inflammation to PPBC progression.
Effects of abrupt involution and its relationship with cancer progression [47]	Mouse model: Wild type FVB/N mice	Abrupt involution leads to dense stroma, altered collagen, inflammation, hormone receptor upregulation, and epithelial hyperplasia/metaplasia.

### Immune modulation from involution to postpartum breast cancer

Mammary gland involution is a highly regulated tissue remodelling process that relies on coordinated interactions between epithelial, stromal, and immune cells [44]. Around 48 h post-weaning, epithelial cell death triggers a cascade of cytokine and protease release, initiating the recruitment of macrophages, neutrophils, and lymphocytes into the mammary stroma [89]. Epithelial cell death triggers the release of damage-associated molecular patterns (DAMPs) and release cytokines such as IL-6, TNFα, and TGF-β, initiating a controlled inflammatory response and promote immune cell infiltration [90]. A key regulator of this process is STAT3, which promotes lysosomal-mediated cell death while simultaneously shaping the inflammatory milieu by upregulating acute-phase genes, cytokines, and COX2 [49]. COX2 converts arachidonic acid into prostaglandins, predominantly PGE2, a lipid mediator that exerts context-dependent effects [85]. At physiological levels, PGE2 promotes debris clearance and resolution of inflammation, but when persistently elevated as seen in aberrant or delayed involution it becomes immunosuppressive [91]. High local PGE2 concentrations inhibit IL-2 synthesis, reduce T-cell receptor signaling, and suppress CD8⁺ cytotoxic and NK cell activity, collectively impairing anti-tumor immunity [92]. Simultaneously, COX2/PGE2 signaling induces lymphangiogenesis and vascular remodelling, facilitating the dissemination of potential tumor cells [93].

Early in involution, macrophages and neutrophils display a pro-inflammatory, debris-clearing M1-like phenotype and secrete MMPs to remodel the ECM [45]. However, long-term activation of TGF-β and PGE2 polarizes macrophages toward an M2-like phenotype, that secretes IL-10, ARG1, and MMP-9, suppressing cytotoxic immunity and promoting collagen deposition and ECM stiffening [91, 94]. Additional regulatory molecules further amplify this process. SEMA7A, which is highly expressed during involution and elevated in PPBC tissues, enhances monocyte chemotaxis, promotes GM-CSF driven macrophage expansion, and reinforces M2-like polarization. SEMA7A-integrin interactions further influence fibroblast activity and collagen organization [95–97]. These changes sustain chronic low-grade inflammation while simultaneously protecting epithelial cells from apoptosis thus creating a permissive environment for survival of transformed or pre-malignant clones. Other emerging regulators include IL-4, IL-13, IL-10, and TGF-β, all of which add to the production of an immunosuppressive environment during involution [90, 98].

A hallmark of late involution is the enrichment of immunosuppressive leukocytes. Regulatory T cells (Tregs) expand in response to TGF-β and hypoxic HIF-1α signaling, while myeloid-derived suppressor cells (MDSCs) infiltrate the gland and inhibit T-cell function [90, 99]. At the same time, epithelial cells and infiltrating myeloid cells may upregulate PD-L1, contributing to T-cell exhaustion. Consistent with this immunosuppressive landscape, transcriptomic profiling of PPBC has revealed distinct immune signatures characterized by altered T-cell activation and differentiation pathways, suggesting that immune remodeling occurring during involution may directly shape the T-cell landscape of PPBC [100]. These suppressive programs, generally short-lived in physiological involution, may persist when involution is delayed or dysregulated [101]. Studies show that the involuting microenvironment shares molecular features with wound healing and fibrosis including elevated IL-6, S100A8/A9, osteopontin, and pro-tumorigenic chemokines such as CCL2 and CXCL12 creating environment that supports dormant tumor cell reactivation or the expansion of newly transformed cells [102–104].

During postpartum involution, these physiological processes may be prolonged or dysregulated, contributing to a microenvironment that has been associated with tumor progression and adverse outcomes in PPBC. Elevated fibrillar collagen and COX2/PGE2 enhance tumor cell invasion and lymphovascular spread. Lymphangiogenesis driven by COX2, VEGF-C/D, and inflammatory macrophages increases metastatic potential. This finding was validated in mouse models, where involution markedly accelerates nodal dissemination. M2-like macrophages and CAFs work together to remodel the ECM. Simultaneously, immunosuppressive cell infiltration by Tregs, MDSCs, and exhausted T cells create a failure of anti-tumor immunity precisely when ductal epithelium is more prone to tumorigenesis.

**Fig. 2:**
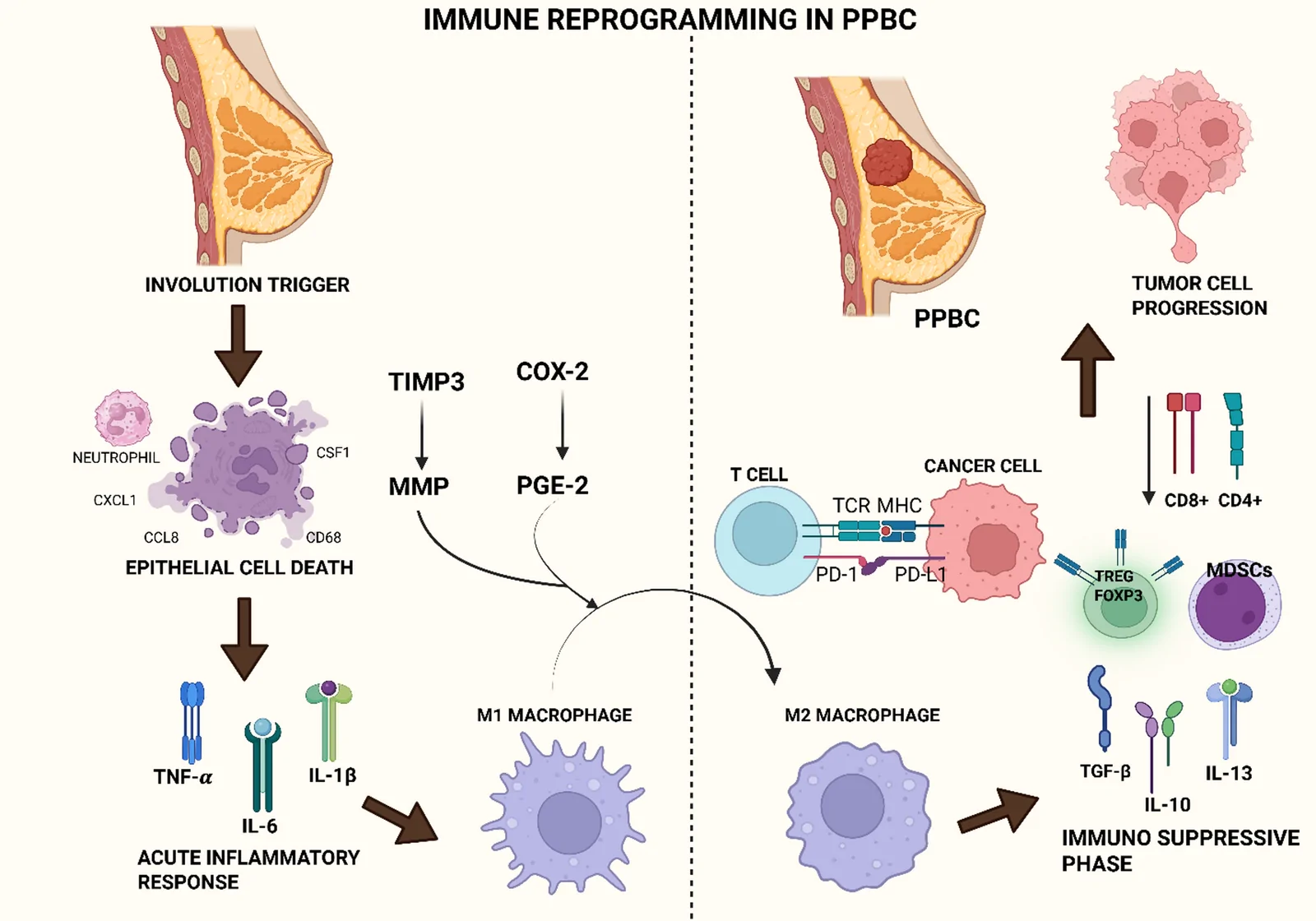
Immune reprogramming in PPBC post lactational involution triggers massive epithelial cell death followed by immune cell infiltration of neutrophils, macrophages and dendritic cells. Activated by TNF-α, IL-1β, and IL-6, the macrophage chemoattractant and their receptors CXCL1, CCL8, CSF1, and CD68 trigger an initial inflammatory response. During PPBC, this proinflammatory environment changes into a chronic immunosuppressive phase, where M1 macrophages polarize into M2 macrophages, releasing anti-inflammatory cytokines like IL-10, TGF-β, and IL-13. The conversion of M1 to M2 macrophages is highly influenced by the upregulation of matrix metalloproteinases (MMPs) and ADAM17, which is regulated by TIMP3, as well as COX2-derived PGE2. This leads to the expansion of FOXP3⁺ Tregs and MDSCs, suppressing antitumor immunity and PD-1/PD-L1 interactions between T cells and tumor cells inducing T cell exhaustion and facilitating immune evasion, collectively promoting tumor cell progression in PPBC. (Created using BioRender.com)

Together, these progressive molecular and cellular events explain how normal postpartum involution, although physiologically necessary, transiently mirrors many hallmarks of a tumor-promoting environment. When involution signals are heightened, prolonged, or coincide with emerging oncogenic lesions, this window becomes uniquely permissive for tumor initiation, immune evasion, and metastasis. The convergence of STAT3, TGF-β, COX2/PGE, SEMA7A, IL-6, and ECM-driven mechanical pathways establishes a chronic, wound-like microenvironment that links the biological program of postpartum involution to the increased risk and aggressive biology of PPBC. Based on the insights from the literature, immune reprogramming that happens during the development of PPBC is depicted in Fig. 2.

### Role of estrogen and its receptor in mammary involution and postpartum breast cancer

The mammary epithelium is highly responsive to fluctuations in circulating sex steroid hormones, and therefore the influence of estrogen and its receptor cannot be excluded in the biology and development of PPBC tumors. Estrogen is a central regulator of mammary gland development, and its actions extend beyond epithelial proliferation to a profound influence on the immune and stromal compartments [105]. During the transition from lactation to involution, estrogen signaling can significantly reprogram the mammary microenvironment. Evidence from recent studies indicates that estrogen and its receptors may modulate immune cell composition, inflammatory responses, and stromal dynamics in ways that could shift the balance toward a pro-tumorigenic state during postpartum involution. However, much of this evidence was using ovariectomized models treated with exogenous estrogen supplements and therefore do not reflect physiological doses as the estrogen levels are likely to be low during phase of involution. These observations may still be of clinical relevance particularly in the context of postpartum exposure to estrogen-containing contraceptives often used after childbirth which may influence the involution-associated microenvironment, although direct evidence in humans remains limited and warrants further investigation [106].

Studies characterizing estrogen-driven immune changes in the normal mammary gland show that estrogen shapes basal immune homeostasis by influencing leukocyte abundance, trafficking, and cytokine expression patterns. Estrogen enhances epithelial expression of chemokines and growth factors that support leukocyte recruitment, especially neutrophils and macrophages, and primes these infiltrating cells toward tissue remodelling phenotypes. During involution, these effects become amplified because the gland is already in a state of increased inflammatory state. Thus, even physiological levels of estrogen may expand neutrophil and macrophage populations that participate in clearing apoptotic cells, restructuring ECM, and releasing soluble mediators associated with wound resolution [106].

Estrogen does not simply increase immune cell numbers, it fundamentally reprograms their activity. Recent evidence demonstrates that estrogen modulates neutrophil function in a way that promotes a pro-tumoral milieu during mammary involution. Estrogen-exposed neutrophils acquire enhanced capacity for ECM remodelling, angiogenic factor secretion, and production of reactive oxygen species [107]. These neutrophils exhibit gene expression signatures consistent with tumor-associated neutrophils, including upregulation of MMPs, VEGF, and inflammatory cytokines. During involution, a period already characterized by high matrix turnover and immune infiltration, estrogen-reprogrammed neutrophils may further enhance wound-healing-like stromal programs by promoting collagen deposition and fibroblast activation, potentially contributing to a microenvironment that supports PPBC progression. This estrogen-neutrophil axis may contribute to an environment in which dormant or newly transformed epithelial cells can expand. In addition, estrogen has been shown to modulate the function of multiple immune cell populations, including macrophages, T cells, and myeloid-derived suppressor cells, influencing processes such as cytokine production, immune suppression, and tissue remodelling. These broader immunomodulatory effects may further shape the tumor microenvironment during involution and contribute to PPBC progression [108–111].

Within the tumor microenvironment (TME), the estrogen pathway influences not only immune cells but also fibroblasts, endothelial cells, and tumor-initiated epithelium. Estrogen receptor (ER) signaling activates transcriptional programs in stromal fibroblasts that increase CXCL12, IL-6, and matrix-modifying enzymes, promoting a chemotactic and pro-survival niche for tumor cells. Stromal ER signaling can also regulate angiogenesis, in part by increasing VEGF production and remodelling perivascular niches. Importantly, studies examining the estrogen pathway in the TME show that ER signaling in non-epithelial compartments can independently modulate tumor progression highlighting that influence of estrogen in PPBC extends beyond classical epithelial ER-driven proliferation [109].

During postpartum involution, these estrogen-mediated stromal effects may synergize with physiological tissue breakdown and remodelling. The involuting gland exhibits elevated fibrillar collagen, COX2 expression, immune infiltration, and lymphangiogenesis, all hallmark features of a tumor-permissive environment. Estrogen can enhance these processes by inducing COX2, expanding neutrophil and macrophage-derived inflammatory mediators, and promoting fibroblast activation. As involution transiently mimics a wound-healing program, ability of estrogen to sustain or amplify inflammatory and stromal remodelling pathways prolong the window of microenvironmental susceptibility, increasing the likelihood of tumor cell survival and dissemination in postpartum tissues [108].

These discoveries regarding actions of estrogen on immune cells in PPBC highlight the potential of anti-estrogen approaches to reprogram the immunosuppressive TME, ultimately strengthening and prolonging responses to current therapies.

### Current therapies, preclinical advances, and ongoing clinical trials in PPBC treatment

Despite substantial evidence suggesting that PPBC differs substantially from BC diagnosed in age-matched non postpartum women, no clinical practice guidelines exist that address its unique biology or therapeutic vulnerabilities. This is perhaps due to lack of concrete evidence from prospective trials that demonstrate different therapeutic options benefit PPBC patients. PPBC patients are currently treated according to standard regimens developed for young women with BC, which may not fully account for the tumor-promoting environment of postpartum mammary involution. More aggressive surgical approaches such as radical mastectomy are commonly pursued in PPBC due to more advanced stage at presentation [12]. Systemic therapies, including neoadjuvant chemotherapy, also show reduced efficacy in this setting. Pathological complete response rates are lower in PPBC than in non-postpartum cases. This disparity might be due to the chemotherapy-induced amplification of inflammatory wound-healing pathways that are already active during involution and may facilitate metastatic dissemination [112]. ER+ PPBC tumors are known to be more aggressive and resistant to endocrine treatment compared to ER+ tumors in age matched non-PPBC tumors [100]. Endocrine therapy is also challenging, as ER+ PPBC often shows a poor response to tamoxifen, potentially due to the hormonal fluctuations that occur after childbirth.

As understanding of postpartum involution increases, it is evident that PPBC requires treatment strategies that extend beyond conventional BC treatment. The involuting mammary gland shows features of a transient yet highly tumor-promoting microenvironment, characterized by intense inflammation, stromal remodelling, immunosuppression, and enriched growth factor signaling as discussed above. Together, these processes reduce the effectiveness of traditional therapies while underscoring the need for new mechanistic targets for intervention. PPBC tumors exhibit an immunosuppressive landscape marked by exhausted CD8 + T cells co-expressing inhibitory receptors like PD-1 and LAG-3, coupled with elevated PD-L1 expression on tumor and immune cells [101]. Preclinical studies in postpartum mammary tumor models demonstrate that anti–PD-1 therapy can reverse the tumor-promoting effects of involution by eliminating exhausted T-cell populations, expanding polyfunctional interferon-γ- and TNFα-producing cytotoxic T cells, and reducing lymphatic vessel density [101]. These findings suggest that immune checkpoint blockade may be particularly effective in PPBC.

Combination therapies that target both the tumor and the involution microenvironment are emerging as a promising option. Chemotherapy paired with nonsteroidal anti-inflammatory drugs (NSAIDs) exemplifies such an approach. Involution is driven by COX2 overexpression, prostaglandin production, and the recruitment of immunosuppressive myeloid cells, factors that collectively enhance metastasis. COX2 inhibition with agents such as ibuprofen has shown the ability to reduce lymphangiogenesis, modify ECM architecture, and restore cytotoxic T-cell activity in preclinical PPBC models [69, 113, 114]. Although NSAIDs are not yet incorporated into clinical management, their low cost, accessibility, and safety profile make them an attractive adjunct for future PPBC specific trials. Supporting this direction, a large Danish cohort study found that low-dose aspirin use was associated with reduced BC recurrence. Given the inflammation-driven biology of PPBC, these findings suggest that low-dose aspirin and other anti-inflammatory agents may help lower recurrence risk, further strengthening the rationale for incorporating anti-inflammatory strategies into combination treatments tailored to the postpartum microenvironment [115]. In line with this an ongoing phase II clinical trial from Mayo clinic is evaluating the effect of low dose aspirin for the prevention of PPBC [116].

The immune landscape of the postpartum breast further highlights macrophages as therapeutic targets. Postpartum involution is dominated by alternatively activated M2-like macrophages that promote collagen deposition, suppress cytotoxic lymphocytes, and facilitate tumor progression [37]. Inhibitors of CSF1R, which controls macrophage recruitment and polarization, could diminish this pro-tumorigenic population [117]. Conversely, agents like CD40 agonists may reprogram M2 macrophages toward a tumoricidal M1 phenotype, counteracting one of the key drivers of progression during involution [118]. In line with this, a study by Eubank et al. based on murine model study reported that granulocyte macrophage colony-stimulating factor (GM-CSF) blocked macrophages VEGF activity by producing soluble VEGF receptor-1 (sVEGFR-1) and showed that GM-CSF treatment slowed tumor growth and metastasis [119]. Another marker, SEMA7A could be an effective therapeutic target for PPBC because it promotes pro-lymphatic macrophages, enhances lymphangiogenesis, and drives metastasis through the lymphatic system, processes that are already enhanced during postpartum involution [86]. In preclinical models of ER + BC, inhibition of SEMA7A using the monoclonal antibody SmAbH1 has shown promising activity both as a single agent and when combined with endocrine therapy. These findings suggest that targeting SEMA7A could help counteract the aggressive, metastasis-prone biology of PPBC and improve treatment responses [120]. Blocking SEMA7A may therefore reduce the aggressive, metastasis-prone behavior of PPBC tumors. Its potential detectability in blood also makes it a useful biomarker for identifying high-risk patients who may benefit from early, intensified treatment [86].

ECM remodelling is another hallmark of postpartum involution that has direct relevance for therapeutic development. Involution involves dense fibrillar collagen deposition, tissue stiffening, and enhanced response to mechanical stress, which collectively create a microenvironment conducive to tumor cell invasion and metastasis. This collagen-rich milieu also activates proteases such as pregnancy-associated plasma protein-A (PAPP-A), which cleaves IGFBP-5, thereby liberating IGF-1 and amplifying proliferative signaling through IGF-1R and DDR2 pathways [121, 122]. Therapeutic strategies aimed at disrupting ECM-dependent tumor promotion include inhibitors of LOX, which mediates collagen cross-linking and tissue stiffening, and small-molecule or antibody-based inhibitors of MMPs, which further remodel the ECM to favor metastasis [123]. Though early in development, such agents may be particularly well-suited to PPBC, where ECM changes are rapid, pronounced, and mechanistically central to disease progression. Recent insights into PAPP-A biology offer additional avenues. Abrupt cessation of lactation intensifies involution heightening inflammation, increasing collagen deposition, and sharply elevating PAPP-A activity thereby boosting IGF signaling. In contrast, gradual weaning has been shown to elevate natural PAPP-A inhibitors such as stanniocalcin-1 and − 2 (STC1/2), which reduce IGF availability and its downstream proliferative effect [121, 122]. This suggests that PAPP-A inhibitors, IGF-1R targeting antibodies, and small-molecule IGF pathway inhibitors could be explored as targeted therapies. Agents that mimic or increase STC1/2 activity may offer an especially appealing, low toxicity strategy that aligns directly with postpartum physiology.

Findings from the KARISMA randomized controlled trial show that low-dose tamoxifen can reduce collagen organization associated with increased tissue stiffness in the normal breast. Since higher collagen density and stiffness are known risk factors that promote tumor initiation and progression, these results suggest that low-dose tamoxifen may help create a less tumor-promoting microenvironment [124]. This raises the possibility that such an approach could serve as a preventive strategy for women at increased risk of developing BC such as PPBC. Ongoing clinical studies are advancing our understanding of PPBC and exploring strategies for its early detection and prevention. As mentioned above ongoing phase II trial is evaluating whether low-dose aspirin can modulate inflammatory markers in postpartum women undergoing breast biopsy, based on the premise that chronic inflammation may elevate PPBC risk [116]. Breast milk has also been explored as a source of tumor-derived biomarkers for monitoring disease recurrence in women previously diagnosed with BC, highlighting its potential as a non-invasive biospecimen for PPBC surveillance [125]. In the ongoing clinical research using multi-omics approaches including genomic, epigenomic, proteomic, and viromic profiling researchers aim to identify molecular signatures that differentiate women who develop PPBC from those who do not [126].

As research advances, digital pathology and artificial intelligence may help drive improvements in PPBC treatment. Quantitative imaging and spatial transcriptomics can capture key features of the postpartum tumor microenvironment such as collagen density, immune infiltration, macrophage phenotypes, or markers of IGF pathway activation and may enable clinicians to identify patients who are most likely to benefit from specific involution-targeting therapies. Machine-learning models that integrate reproductive history, lactation behaviors, tumor genomics, and microenvironmental signatures may further improve risk stratification and guide individualized therapeutic choices. These tools are especially relevant for PPBC, where reproductive history is both a clinical and mechanistic variable shaping disease trajectory.

**Fig. 3:**
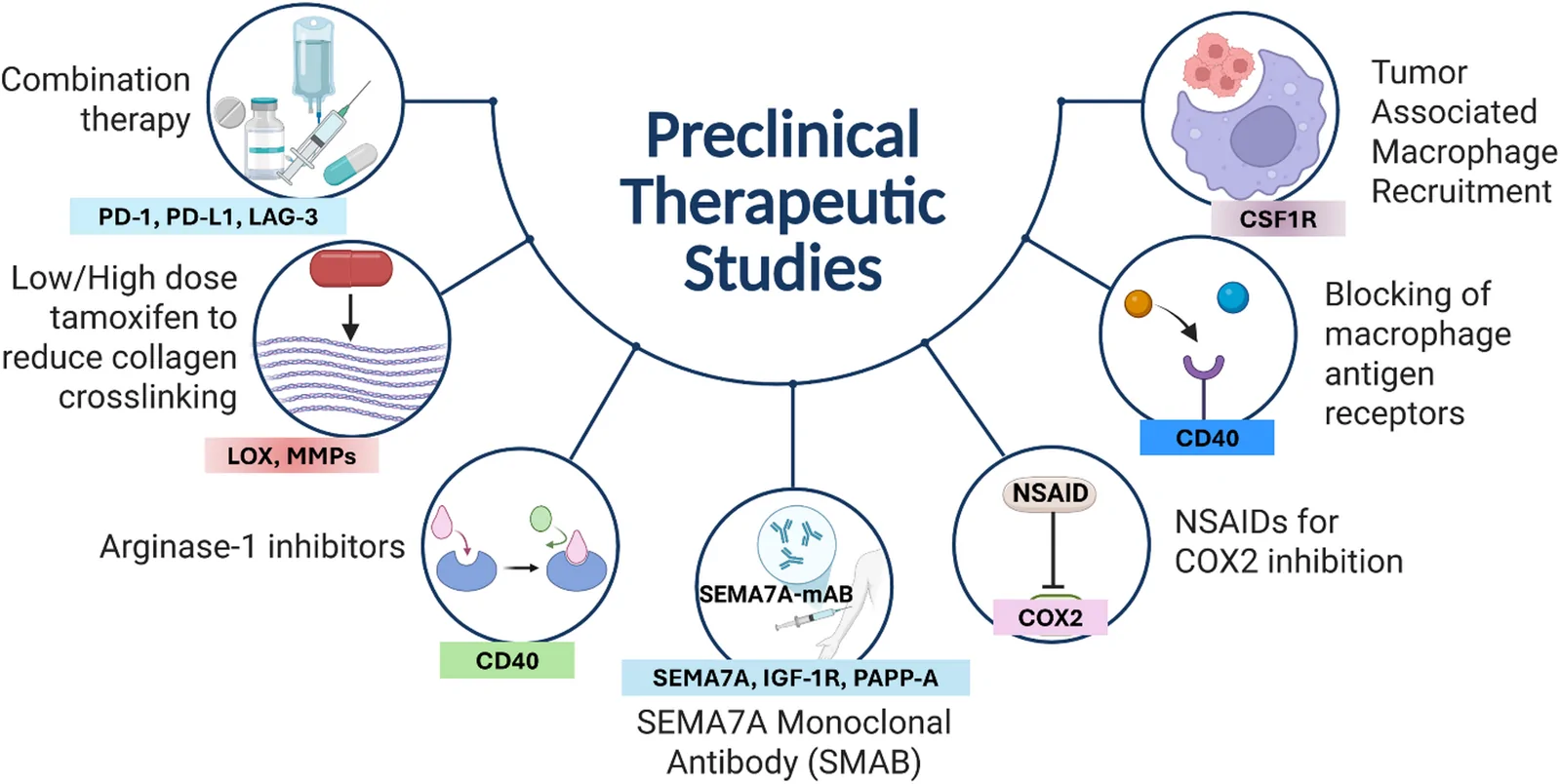
Preclinical therapeutic studies: a schematic illustration of preclinical therapeutic options in PPBC, focusing on key molecules and pathways are COX2 inhibition, low dose tamoxifen, macrophage blocking, and impeding SEMA7A signaling and use of non-steroidal anti-inflammatory drugs (NSAIDs). (Created using Biorender.com)

Effective PPBC treatment should support the whole person, including their emotional health and their needs around fertility and family. Given the long post-pregnancy window during which PPBC risk remains elevated, enhanced surveillance protocols for women within 10 years postpartum may facilitate earlier detection. Multidisciplinary care that includes fertility preservation counseling, psychological services, and discussions about reproductive planning should be standard, as these considerations significantly influence treatment decisions and quality of life. Rapid diagnosis and early initiation of therapy are important, as the unique postpartum microenvironment can accelerate tumor growth and spread. Collectively, these pathways highlight multiple actionable molecular targets within the postpartum tumor microenvironment, including immune checkpoints (PD-1/PD-L1, LAG-3), inflammatory mediators (COX-2), macrophage regulators (CSF1R, CD40), ECM remodeling enzymes (LOX, MMPs), and growth factor signaling components (IGF-1R, PAPP-A, SEMA7A), as summarized in Fig. 3, and these promising preclinical therapeutic insights should inform the design of prevention and treatment clinical trials specifically for PPBC, ultimately supporting the adaptation of clinical BC treatment guidelines.

## Conclusion

PPBC represents a clinically and biologically distinct subtype of BC arising during a highly vulnerable window after childbirth. Recent literature suggests discontinuing the use of the term PABC, allowing researchers to focus more precisely on PrBC and PPBC. This distinction may enable more tailored and effective treatment strategies, particularly for PPBC, which is associated with a poorer prognosis. Evidence produced across epidemiology, mammary gland biology, ECM remodelling, immune modulation, and hormonal signalling clearly demonstrates that postpartum involution creates a wound-healing–like microenvironment that inadvertently supports tumor initiation and metastasis. Dysregulated involution, aberrant estrogen and STAT3 signalling, ECM stiffening, COX2/PGE2 driven inflammation, macrophage reprogramming, and lymphangiogenic remodelling collectively shape a uniquely aggressive tumor ecosystem that cannot be fully addressed by standard subtype-based therapies. Importantly, recent efforts to translate mechanistic discoveries into clinical benefit are underway. Early-phase trials including low-dose aspirin for inflammation modulation, COX2 inhibitors, immune-microenvironment targeted studies, and endocrine-response profiling tools represent a major step toward PPBC-specific management. Yet significant gaps remain in developing validated biomarkers, defining optimal postpartum risk windows, and designing targeted therapies for this high-risk population.

To truly improve outcomes, PPBC must be treated as a separate clinical entity in research, diagnosis, and hopefully treatment in the future. Prospective work integrating spatial biology, multi-omics profiling, ECM-targeted therapy, immunomodulation, and precision endocrine strategies will be critical. A multidisciplinary approach that unites basic biology, clinical oncology, obstetrics, and survivorship care is urgently needed to bring PPBC-specific therapeutic interventions into routine clinical practice and meaningfully reduce the global burden of this overlooked and aggressive disease.

## Data Availability

All data relevant to this review are included within the manuscript.
